# The Relationship of HLA Class I and II Alleles and Haplotypes with Autism: A Case Control Study

**DOI:** 10.1155/2014/242048

**Published:** 2014-02-03

**Authors:** Manan Al-Hakbany, Sitalbanat Awadallah, Laila AL-Ayadhi

**Affiliations:** ^1^College of Medicine, King Saud University, P.O. Box 2925, Riyadh 11461, Saudi Arabia; ^2^Autism Research and Treatment Center, Al-Amodi Autism Research Chair, Saudi Arabia

## Abstract

Earlier reports showed the relationship between autism and immune genes located in the human leukocyte antigen (HLA). In this current study, we compared the HLA class I and class II alleles and haplotypes in 35 autistic children with 100 control subjects from Saudi Arabia, using PCR-SSP method and Luminex technology. In class I the HLA-A*01 (*P* = 0.03, OR 2.68), A*02 (*P* = 0.001, OR 3.02) and HLA-B*07 (*P* = 0.01, OR 3.27), were significantly associated with autism. Also, the haplotype A*02-B*07 was significantly higher in autistic patients than in controls (*P* = 0.007, OR 5.83). In class II, DRB1*1104 was significantly higher in patients than in controls (*P* = 0.001, OR 8.75). The DQB1*0202 (*P* = 0.001, OR 0.24), DQB1*0302 (*P* = 0.001, OR 0.14), and DQB1*0501 (*P* = 0.012, OR 0.25), were negatively associated with disease. While the four-loci genotype study showed that A*01-B*07-DRB1*0701-DQB1*0602 (*P* = 0.001, OR 41.9) and the A*31-B*51-DRB1*0103-DQB1*0302 (*P* = 0.012, OR 4.8) are positively associated with autism among Saudi patients. This is the first report on a foreseeable risk of association of HLA-B*07 allele with autism. Thus, HLA-B*07 allele and the closely linked haplotype A*01 B*07 DRB1*0701 DQB1*0602 may serve as a marker for genetic susceptibility to autism in Saudis.

## 1. Introduction

Autism spectrum disorders (ASD) are neurodevelopmental syndromes characterized by early childhood onset, associated with brain abnormalities [1]. ASD includes autistic disorder, Asperger's syndrome, Rett syndrome, childhood disintegrative disorder (CDD), and pervasive developmental disorders not otherwise specified (PDD-NOS), as per the DSM-IV-TR classification according to American Psychatric Association, 2000.

The incidence of ASD has dramatically risen in the past two decades affecting 1 in 88 children (about 1 in 54 boys and 1 in 252 girls) in the United States [2]. Broader diagnostic criteria and increased medical knowledge have contributed to this perceived increase in disease incidence [3].

The aetiology of ASD is still unclear; nevertheless, both genetic and environmental causes are believed to contribute to the risk for the development of this disease spectrum. More evidence suggests that environmental factors, such as exposure to toxic compounds, teratogens, perinatal insults, and prenatal infections, may be responsible directly or indirectly for the immune mechanisms that mediate the nervous system impairments seen in ASD [4, 5].

Family studies uncovered a recurrence risk to siblings of ASD children within the range of 2–6%, based on a higher concordance between monozygotic twins (36–91%), compared to a 10% concordance in dizygotic twins [6]. Also, risk of ASD is much higher in families than in the general population but lower than what would be expected in single-gene diseases [7, 8]. The wide phenotypic variability of ASD suggests that diverse genes, gene-gene interactions, and gene-environment interactions play a vital role in this disease [9, 10].

HLA genes are part of the major histocompatibility complex (MHC) localized on the short arm of chromosome 6. High levels of polymorphisms characterize MHC genes. These genes are directly involved in immune response and related to many other autoimmune diseases such as type 1 diabetes [11].

The possible associations between (HLA) alleles and autism have received wide coverage from different ethnic background [12–14]. In particular, case-control and transmission disequilibrium test (TDT) analyses have suggested that HLA-DR4 increase the susceptibility to autism, whereas HLA-DR13 has a protective role toward disease development [15]. However, studies that used sib-pair study did not support HLA association [16]. Subsequent studies [17–19] reinforced earlier findings of the role of an extended haplotype in ASD incidence. They reported that 44.1 haplotype occurs more frequently in autism than controls [20, 21]; this haplotype contains DR*β*1*04 (DR4) in class II region and A2 and B44 alleles in class I region. The HLA-A and HLA-B are in linkage disequilibrium with different genetic loci in the two different polymorphic blocks [22]. Thus, any gene within these regions may be associated with ASD. In addition, Boulanger and Shaz shown that HLA class I molecules play a vital role in brain development [23].

The main aim of this study is to find out the link between HLA class I and class II with autism in Saudi autistic children compared with normal controls.

## 2. Subjects and Method

### 2.1. Study Population

35 children (12 females and 23 males; mean age: 6 years) with a diagnosis of ASD according to the 4th edition of the diagnostic and statistical manual of mental disorders criteria [24]. Patients recruited from the Autism Research and Treatment Centre, Al-Amodi Autism Research Chair, Department of Physiology, King Saud University, Riyadh, Saudi Arabia. Patients who were associated with neurological disease (such as palsy and tuberous sclerosis) and metabolic disorders (e.g., phenylketonuria) were excluded from the study.

The control group consisted of 100 unrelated healthy Saudi adults who were recruited and screened (at King Faisal Specialist Hospital) for HLA class I and class II as potential bone marrow donors.

This study received approval from the Ethical Committee of King Khalid University Hospital. Patients' parents gave informed consent prior to inclusion in the study.

### 2.2. Genotyping

Genomic DNA was isolated from peripheral blood by phenol chloroform extraction using Qiagen QIAamp DNA blood kit.

### 2.3. HLA Polymorphism

HLA typing, of class I HLA-A, HLA-B, and HLA-C and class II DRB1 and DQB1 loci, was performed by standard sequence specific primer polymerase chain reaction (SSP-PCR).

### 2.4. HLA Typing

The target DNA was amplified by PCR using a group-specific primer. The biotinylated PCR product allows it to be detected using R-Phycoerythrin-conjugated Strepavidin (SAPE). A flow analyzer (Luminex) identifies the fluorescent intensity of PE (phycoerythrin) on each microsphere. The assignment of the HLA typing based on the reaction pattern was compared to patterns associated with published HLA gene sequences. HLA Fusion software by One Lambda Inc. is used to analyze the reaction pattern.

### 2.5. Statistical Analysis

The significance of difference in frequencies of HLA-A, -B, HLA-C, DQB1, and DRB1 alleles between patients and controls was compared by Chi-square (*χ* ^2^) test with Yates correction and Fisher's exact test. Odds ratio (ORs) and 95% confidence intervals (CIs) were calculated to determine levels of significances. For all tests, a probability (*P*) of less than 0.05 was significant.

## 3. Results

The frequencies of HLA class I and class II of 35 autistic patients and 100 healthy controls showed in Tables 1 and 2. They include all alleles at frequencies not less than 1%. HLA alleles frequencies were shown as 2*n* level in both patients and controls.

In HLA class I HLA-A*01 (*P* = 0.03, OR 2.68), HLA-A*02 (*P* = 0.001, OR 3.02) and HLA-B*07 (*P* = 0.01, OR 3.27) alleles were found to be significantly associated with autism. None of the HLA-C alleles frequencies shows any significant association with autism among Saudi patients (Table 1).

Analysis of HLA DRB1 and DQB1 showed that only DRB1*1104 was significantly higher in patients than in controls (*P* = 0.001, OR 8.7). On the other hand, neither DRB1*04 nor DRB1*03 has as association with autism in this study. Three of the DQB1 alleles are negatively linked to autism such as DQB1*0202 (*P* = 0.001, OR 0.24), DQB1*0302 (*P* = 0.001, OR 0.14), and DQB1*0501 (*P* = 0.012, OR 0.24).

The analysis of A*-B* haplotypes showed that HLA-A*02-B*07 haplotype associated significantly with autism (*P* = 0.007, OR 5.83); data was not shown.

The HLA-A-B-DRB1-DQB1 genotype association with autism showed in Table 3. The following haplotypes were significantly higher among autistic patients than controls: A*01 B*07 DRB1*0701 DQA1*0602 (*P* = 0.001, OR 41.9) and A*31 B*51 DBB1*0103 DQB1*0302 (*P* = 0.01, OR 24.8).

## 4. Discussion

Although the etiopathogenic mechanism(s) of autism is not clear, genetic and environmental factors are believed to play a role in the onset of the autism [25]. However, recently both genetic and environmental interdependence studies came in support of a pivotal role for immune-related genes and immune responses to environmental stimuli [26, 27]. In particular, many of the proteins encoded by the major histocompatibility complex (MHC) showed to be closely linked with the formation, refinement, maintenance, and plasticity of the brain [28, 29]. Furthermore, there is an emerging concept suggesting that disruptions in MHC expression in the developing brain caused by mutations and/or immune deregulation may contribute to the altered brain connectivity and function characteristic of autism [30].

At present, there is no consensus on the HLA link with autism susceptibility and protection and large inconsistent results exist between the findings of different studies. One of the studies which prove the association of HLA with autism claims that transmission disequilibrium test results suggested that DR4 and DR13 were linked to ASD [15]. Another study from Egypt showed that there is a positive association between DRB1*11 allele and autism, and also a protective function assigned to DRB1*03. From the same study, an increased disease risk was found among families with history of autoimmune disease [31]. Furthermore, a study from china found that DR4, DR11, and DR14 had a different effect on intelligence and neuropsychology tests among autistic children [32], unlike an earlier study, which shows no significant association between autism and HLA [33].

In the present study, using the PCR-SSP method, HLA-A, B, C, DRB1, and DQB1 were screened in high resolution (Tables 1 and 2). The findings obtained have shown that HLA-A*1 and *02 alleles are significantly associated with autism. In previous studies HLA-A2 allele was associated with other autoimmune diseases, such as juvenile rheumatoid arthritis [34] and Hashimoto's thyroiditis [35]. While other studies have found an excess of HLA-A2 in Alzheimer's disease, a neurodegenerative condition associated with inflammatory signs [36, 37]. In addition, HLA-A2 allele may play a prominent role as a restricting factor in cytotoxic T-cell recognition in the feto-maternal relationship mainly to male fetuses [38].

In the present study the HLAB*07 is the allele that is significantly associated with autism. This original association is not reported before in other populations. Also A2-B*07 haplotype is significantly associated with the disease. This is unlike the previous finding of Torres et al. who report a higher prevalence of A2-B44 and A2-B51 among autistic children than controls [13].

The A*01-B*07 and A*02-B44 haplotypes are known to be associated with autism in some of the previous studies [13], but in the present study their frequencies in autistic patients are not different from controls. The A*02-B*44 haplotype is part of the larger B44-SC30-DR4 extended haplotype, which is more frequent in autistic children than in controls [14, 20]. This extended haplotype also contains two genetic loci previously shown to be associated with autism, the C4B null allele, and HLA-DR4 [15, 21, 39].

In class II, we found a positive association between DRB1*11 allele and autism, similar to the findings of a study from Egypt [31]. In the present study there was no association between DR4 and autism, in contrast to positive association of DR4 allele with autism from a study done in Caucasian [15, 40].

In the current study, the DQB1*0202, *0302, and *0501 alleles are significantly higher among controls than patients. Unlike Mostafa et al. who reported a negative association of DR3 with autism [31]. In another study, DR 13 was found to confer protection from autism in a study on Caucasian population [15].

Analysis of the four loci haplotypes that are associated with autism showed that the A*01-B*07-DRB1*0701-DQAB1*0602 has the highest disease risk (OR 41.15). This haplotype includes the significant risk alleles A*01 and B*07 which makes them valuable markers for autism among Saudis. Also the A*31-B*51-DRB1*0103-DQB1*0302 (OR 4.8) is considered a risk haplotype. The B51 allele frequency in patients was twice as higher than controls; similarly the A2-B51 haplotype frequency is higher among patients than in controls, but those associations are not statistically significant.

Genetic studies unavoidably raise the attention to the ethnic variation in the incidence of disease. There are many possible explanations for the above-stated variation in HLA associations with autism across different populations. The important explanation involves the interaction between HLA allele and different infectious agents or environmental allergen across geographical regions. Nevertheless the role of ethnic differences on HLA allele frequencies contributed also to this variation. It must be emphasised that HLA haplotype determination remains of interest for clarification of susceptible and resistance genes to autism [19].

In the current study of HLA association with autism among Saudi children has the following positive association between HLA*A01, HLA*A02 HLA*B07, HLA DRB1*11 alleles, and A*01-B*07-DRB1*0701-DQB1*0602; A*31-B*51-DRB1*0103-DQB1*0302 haplotypes demonstrate their involvement in the disease aetiology possibly by playing a role in the presentation of microbial antigen within the central nervous system, which may interfere with the formation of synaptic and neuronal circuits in the developing brain.

Studies associating autism with HLA class I and class II are scarce and have generated contradictory findings. This may be attributed to the complexity of the disease spectrum and a small number of cases studied. However, the increasing incidence of autism will enable further studies which will provide better understanding of aetiopathogenesis of autism.

In conclusion despite a relatively small sample size, this research reports and for the first time a foreseeable risk association of HLA-B*07 allele and the closely linked haplotypes A*01 B*07 DRB1*0701 DQB1*0602 might serve as genetic markers for susceptibility to autism in Saudis.

## Figures and Tables

**Table 1:** HLA-A*, -B*, and -C* allele frequencies in autistic children and controls.

HLA	Autism	Controls	Statistical analysis	
	2n = 70 *N* (%)	2n = 200 *N* (%)	OR (95% CI)	*P*
A*01	**12 (17.6)**	**15 (7.50)**	**2.68 (1.08–6.42)**	**0.03**
A*02	**30 (42.8)**	**54 (27.00)**	**3.02 (1.56–5.83)**	**0.001**
A*11	2 (2.9)	7 (3.50)	0.83 (0.11–4.54)	1.00
A*23	2 (2.9)	12 (6.00)	0.47 (0.07–2.3)	0.51
A*26	6 (8.8)	10 (5.00)	1.8 (0.56–5.71)	0.34
A*29	2 (2.9)	4 (2.00)	1.48 (0.18–9.71)	1.00
A*31	8 (11.8)	12 (6.00)	2.1 (0.73–5.81)	0.19
A*32	2 (2.9)	10 (5.00)	0.51 (0.08–2.9)	0.71
A*33	4 (5.9)	8 (4.00)	1.5 (0.36–5.7)	0.75
A*68	4 (5.9)	20 (10.00)	0.56 (0.15–1.83)	0.43
B*07	**10 (14.7)**	**10 (5.00)**	**3.27 (1.19–9.03)**	**0.01**
B*08	4 (5.90)	12 (6.00)	0.97 (0.25–3.4)	1.00
B*14	4 (5.90)	3 (1.50)	4.1 (0.75–28.31)	0.12
B*15	4 (5.90)	12 (6.00)	0.97 (0.25–3.43)	1.00
B*18	4 (5.90)	7 (3.50)	1.73 (0.4–6.8)	0.60
B*35	2 (2.90)	16 (8.00)	0.34 (0.05–1.6)	0.24
B*40	2 (2.90)	3 (1.50)	1.94 (0.22–15.0)	0.81
B*41	4 (5.90)	9 (4.50)	1.32 (0.33–4.9)	0.85
B*44	2 (2.90)	4 (2.00)	1.48 (0.18–9.7)	1.00
B*50	8 (11.80)	28 (14.00)	0.82 (0.32–2.1)	0.79
B*51	16 (23.50)	42 (21.00)	1.15 (0.57–2.3)	0.78
B*52	4 (5.90)	6 (3.00)	2.01 (0.46–8.4)	0.47
B*57	2 (2.90)	3 (1.50)	2.01 (0.46–8.1)	0.47
B*58	2 (2.90)	7 (3.50)	0.85 (0.11–4.5)	1.00
C*01	2 (2.90)	4 (2.00)	1.48 (0.18–9.7)	1.00
C*04	4 (5.90)	25 (12.50)	0.43 (0.12–1.3)	0.19
C*05	2 (2.90)	2 (1.00)	3.0 (0.29–30.5)	0.57
C*06	12 (17.60)	31 (15.50)	1.16 (0.52–2.56)	0.82
C*07	12 (17.60)	43 (21.50)	0.78 (0.36–1.66)	0.61
C*08	4 (5.90)	4 (2.00)	3.1 (0.62–15.0)	0.22
C*12	6 (8.80)	16 (8.00)	1.11 (0.37–3.2)	1.00
C*15	16 (23.50)	32 (16.00)	1.6 (0.77–3.3)	0.22
C*16	4 (5.90)	10 (5.00)	1.1 (0.32–4.3)	1.00
C*17	6 (8.80)	10 (5.00)	1.84 (0.57–5.7)	0.33

AF: allele frequency; OR: odds ratio; 95% CI: confidence interval; NS: not significant. 2*n*: each individual was represented by two codominant allelic data. The bold font in table refers to significant association of alleles and haplotype with autism.

**Table 2:** The HLA-DRB1* and DQB1* alleles frequencies in autistic children and controls.

HLADR	Autism	Controls	Statistical analysis	
	2n = 70 N (%)	2*n* = 200 N (%)	OR (95% CI)	P
DRB1*0102	4 (5.89)	5 (2.50)	2.4 (0.38–10.8)	0.34
DRB1*0301	10 (14.7)	26 (13.00)	1.15 (0.48–2.6)	0.80
DRB1*0403	4 (5.89)	13 (6.50)	0.89 (0.23–3.1)	1.00
DRB1*0701	10 (14.7)	33 (16.50)	0.87 (0.37–1.9)	0.80
DRB1*1101	6 (8.82)	7 (3.50)	2.66 (0.7–9.2)	0.50
DRB1*1104	**8 (11.76)**	**3 (1.50)**	**8.7 (2.0–43.1)**	**0.001**
DRB1*1301	6 (8.82)	15 (7.50)	1.19 (0.3–3.4)	0.90
DRB1*1302	4 (5.89)	11 (5.50)	1.07 (0.27–3.8)	1.00
DRB1*1501	4 (5.89)	11 (5.50)	1.07 (0.27–3.8)	1.00
DRB1*1502	4 (2.94)	6 (3.00)	0.98 (1.13–5.5)	1.00
DRB1*1601	4 (5.89)	8 (4.00)	1.5 (0.36–5.7)	0.70
**DQB1**				
DQB1∗0201	10 (14.7)	51 (25.50)	0.5 (0.2–1.11)	0.09
** DQB1∗0202**	**8 (11.76)**	**72 (36.00)**	**0.24 (0.09–0.58)**	**0.001**
DQB1∗0301	14 (20.59)	37 (18.50)	1.14 (0.5–2.3)	0.80
** DQB1∗0302**	**4 (5.88)**	**59 (29.50)**	**0.14 (0.04–0.45)**	**0.001**
DQB1∗0303	2 (2.94)	8 (4.50)	0.72 (0.10–3.8)	0.97
DQB1∗0402	2 (2.94)	8 (4.50)	0.72 (0.10–3.8)	0.97
** DQB1∗0501**	**4 (5.88)**	**40 (20.00)**	**0.25 (0.07–0.77)**	**0.012**
DQB1∗0502	4 (5.88)	22 (11.00)	0.5 (0.14–1.6)	0.30
DQB1∗0503	2 (2.94)	4 (2.00)	1.4 (0.18–9.7)	1.00
DQB1∗0601	2 (2.94)	13 (6.50)	0.4 (0.06–2.11)	0.40
DQB1∗0602	4 (5.88)	25 (12.50)	0.4 (0.12–1.3)	0.19
DQB1∗0603	8 (11.76)	35 (17.50)	0.6 (0.25–1.5)	0.35
DQB1*0604	2 (2.94)	16 (8.00)	0.34 (0.05–1.6)	0.24

AF: allele frequency; OR: odds ratio; 95% CI: confidence interval; NS: not significant. 2*n*: each individual was represented by two codominant allelic data. The bold font in table refers to significant association of alleles and haplotype with autism.

**Table 3:** Frequency of HLA haplotypes in autistic children and controls.

Haplotypes				Patients	Controls	Statistical analysis	
HLA class I		HLA class II		no./35 (%)	no./100 (%)	OR (95% CI)	*P*
A*01	B*07	DRB1*0701	DQB1*0602	**5 (14.3)**	**4 (0.50)**	**41.9 (4.9–903.7)**	**0.001**
A*01	B*08	DRB1*0301	DQB1*0201	1 (2.85)	4 (0.50)	6.18 (0.4–179.7)	0.32
A*02	B*08	DRB1*0301	DQB1*0201	1 (2.85)	12 (1.5)	2.02 (0.22–15.8)	0.80
A*02	B*14	DRB1*0102	DQB1*0507	2 (5.71)	7 (0.88)	6.5 (0.9–54.4)	0.058
A*02	B*15	DRB1*1104	DQB1*0301	2 (5.71)	8 (1.00)	6.5 (0.9–54.4)	0.058
A*02	B*18	DRB1*0701	DQB1*0202/06	2 (5.71)	7 (0.88)	6.5 (0.9–54.4)	0.058
A*02	B*35	DRB1*0403	DQB1*0302	1 (2.85)	17 (2.13)	1.5 (0.18–10.2)	1.00
A*02	B*40	DRB1*1101	DQB1*0301	1 (2.85)	5 (0.63)	6.1 (0.41–178.7)	0.32
A*02	B*41	DRB1*1301	DQB1*0603	2 (5.71)	11 (1.38)	4.3 (0.7–26.1)	0.12
A*02	B*44	DRB1*1104	DQB1*0301	1 (2.85)	4 (0.50)	6.1 (0.4–179.7)	0.32
A*02	B*50	DRB1*1102	DQB1*0501	1 (2.85)	42 (5.26)	0.51 (0.07–2.62)	0.59
A*23	B*50	DRB1*1501	DQB1*0603	1(2.85)	18 (2.26)	1.18 (0.15–7.4)	1.00
A*26	B*51	DRB1*1407	DQB1*0503	2 (5.71)	6 (0.75)	6.5 (0.9–54.4)	0.058
A*31	B*51	DRB1*0103	DQB1*0302	**4 (11.42)**	**22 (2.76)**	**4.8 (1.35–17.5)**	**0.01**
A*32	B*51	DRB1*1302	DQB1*0604	1 (2.85)	11 (1.38)	2.02 (0.22–15.8)	0.80
A*68	B*58	DRB1*1201	DQB1*0501	1 (2.85)	5 (0.63)	3.06 (0.29–32.08)	0.57

AF: allele frequency; OR: odds ratio; 95% CI: confidence interval; NS: not significant. The bold font in table refers to significant association of alleles and haplotype with autism.
